# CAR-T cells targeting fibroblast activation protein eliminate pathological fibroblasts and preserve cardiac function in a Duchenne Muscular Dystrophy murine model

**DOI:** 10.1186/s13287-026-05025-1

**Published:** 2026-04-30

**Authors:** Céline Marigny, Gaëlle Revet, Anne Berger, Morgane Boulch, Nathalie Mougenot, Zhenlin Li, Béatrice Corre, Mégane Lemaitre, Axelle Bois, Clara Castelli, Victor Collombat, Ara Parlakian, Marie-Cécile Perier, Adrian Bot, Jonathan A. Epstein, Peggy Lafuste, Albert Hagege, Haig Aghajanian, Clément Cochain, Philippe Bousso, Onnik Agbulut, Philippe Menasché

**Affiliations:** 1 INSERM UMRS 970, Paris Centre de Recherche Cardiovasculaire (PARCC), Univ Paris-Cité, Paris, France; 2 Institut de Biologie Paris-Seine (IBPS), CNRS UMR 8263, Inserm U1345, Development, Adaptation and Ageing, Sorbonne Université, 7, quai St Bernard (case 256), 75005 Paris, France; 3 Dynamics of Immune Responses Unit, Institut Pasteur, Université Paris Cité, INSERM U1223, Paris, France; 4 Sorbonne Université, UMS28, Plateforme d’Expérimentation Cœur, Muscles, Vaisseaux, Paris, France; 5 Capstan Therapeutics, San Diego, CA USA; 6 Department of Cell and Developmental Biology, Perelman School of Medicine, University of Pennsylvania, Philadelphia, PA USA; 7 Penn Cardiovascular Institute, Perelman School of Medicine, University of Pennsylvania, Philadelphia, PA USA; 8 Institute for Regenerative Medicine, Perelman School of Medicine at the University of Pennsylvania, Philadelphia, PA USA; 9 Department of Medicine, Perelman School of Medicine at the University of Pennsylvania, Philadelphia, PA USA; 10 IMRB, Inserm U955-Team Relaix, Université Paris Est-Créteil, Créteil, France; 11 Department of Cardiology, Hôpital Européen Georges Pompidou, Paris, France; 12 Institute of Experimental Biomedicine, University Hospital Würzburg, Würzburg, Germany; 13 Department of Cardiovascular Surgery, Hôpital Européen Georges Pompidou, 56 rue Leblanc, 75015 Paris, France

**Keywords:** CAR-T cells, Fibrosis, Duchenne Muscular Dystrophy, Fibroblast Activation Protein, Cell therapy

## Abstract

**Background:**

Chimeric Antigen Receptor (CAR)-T cells therapy has revolutionized the treatment of hematological cancers and are currently redirected towards non-malignant diseases. If correction of the gene defect remains the cornerstone of the treatment of Duchenne Muscular Dystrophy (DMD), the disease-associated fibrosis can limit its efficacy. We thus assessed the effects of eliminating cardiac fibrosis of DMD by CAR-T cells targeting Fibroblast Activation Protein (FAP), a protein strongly expressed by activated fibroblasts.

**Methods:**

In vitro CAR-T cells expressing both FAP and a green fluorescent probe (GFP) were first co-cultured with FAP + of FAP- target cells to check for FAP-expressing lymphocyte activation. Then, anti-FAP CAR-T cells were intravenously delivered in a dystrophic murine model (D2.*mdx*), following lymphodepletion, to investigate the kinetics, biodistribution, cardiac functional and anti-fibrotic effects of anti-FAP CAR-T cells compared with control lymphocytes engineered to only express GFP. The mechanism of action at a cellular level was assessed by single-cell RNA-sequencing of harvested hearts.

**Results:**

In vitro anti-FAP CAR-T cells were successfully activated when co-cultured with FAP + target cells. In a dystrophic murine model (D2.*mdx*), anti-FAP CAR-T cells, intravenously delivered following lymphodepletion, homed to the heart and skeletal muscles, where they decreased FAP and fibrosis-associated genes. Single-cell RNA-sequencing linked these changes to a decrease in a definite cluster of fibrogenic fibroblasts. Concomitantly, anti-FAP CAR-T cells improved cardiac function compared to control mice injected with GFP-transduced T lymphocytes or bovine serum albumin used as negative controls.

**Conclusions:**

These results suggest that anti-FAP CAR-T cells could be efficient for mitigating fibrosis and thus complement gene therapy of DMD. More generally, their therapeutic benefits pave the way for potential applications extending to other fibrosis-associated diseases.

**Supplementary Information:**

The online version contains supplementary material available at 10.1186/s13287-026-05025-1.

## Background

Chimeric Antigen Receptor (CAR)-T cells therapy has revolutionized the treatment of hematological malignancies through its ability to recognize and kill targeted cancer cells, leading to several clinically approved products to date, with durable complete remissions in large patient populations. These successful outcomes have prompted numerous research teams to explore the potential of this therapy beyond hematological applications, particularly solid cancers and autoimmune diseases [1]. 

Another promising indication is cardiac fibrosis, as suggested by the therapeutic effects of CAR-T cells directed against Fibroblast Activation Protein (FAP), a protein expressed by activated fibroblasts. It is present in the tumoral stroma, where it can hinder the access of CAR-T cells to their cancerous targets [2] but is also upregulated in hypertrophied and dilated hearts. In a pioneering study, Aghajanian et al. [3] have shown in a mouse model of hypertensive cardiomyopathy that CAR-T cells targeting FAP (anti-FAP CAR-T cells) could mitigate fibrosis and improve cardiac function. The pathogenic role of FAP + cells is further supported by the observation, based on ^68^ Ga-FAP inhibitor (FAPI)-46 PET/CT, [4] that in patients with an acute myocardial infarction, the magnitude of the FAP signal is associated with a subsequent impairment of cardiac function. The present study was therefore designed to assess whether the putative benefits of anti-FAP CAR-T cells could also be relevant to the mitigation of fibrosis in Duchenne Muscular Dystrophy (DMD).

DMD is the most common X-linked inherited disease, caused by mutations in the dystrophin gene [5]. Dystrophin is part of a protein complex involved in force transmission and sarcolemmal stability in muscle. Its deficiency leads to an acute sensitivity to mechanical stress inducing damage and inflammation of muscle fibres, increased accumulation of fibro-adipogenic progenitors leading to fibro-calcification of muscle and failure of myogenesis [6]. Ultimately, contractile fibres are replaced by fibrotic and adipose tissue and fibrosis is actually the only pathologic parameter of muscle tissue that correlates with functional outcomes. While fibrosis progressively infiltrates skeletal muscle, leading to locomotor disabilities in early childhood, its impact on heart function contributes to the development of a dilated cardiomyopathy which currently represents the major cause of death in DMD patients within their third or fourth decade [7, 8]. Thus, the impact of fibrosis on both quality of life and life expectancy provides a strong rationale for antifibrotic therapeutic strategies [9].

While gene therapy remains the cornerstone of DMD management, mitigating the associated fibrosis could thus be an effective adjunct for both improving muscular function and facilitating the access of gene products to the diseased myocytes [10, 11]. So far, none of the tested anti-fibrotic therapies have unequivocally shown efficacy, leaving space for newer options. Among them, anti-FAP CAR-T cells are garnering a growing interest, further reinforced by a recent study showing, in young (3 months of age) DMD mice, that anti-FAP CAR-T cells can successfully mitigate fibrosis in skeletal muscles [11]. However, given the strong prognostic implications of heart disease in these patients, the present study more specifically focused on the effects of this adoptive immunotherapy on cardiac function.

To address this question, we selected the D2*.mdx* mouse model. Resulting from a backcrossing of the *mdx* mutation onto the DBA/2J genetic background, D2.*mdx* mice feature a greater fibrotic progression in the heart due to a polymorphism in *Ltbp4*, which is associated with increased SMAD signalling. As such, the D2.*mdx* model is usually considered to more closely mimic the human DMD phenotype than the classic B10.*mdx* dystrophic model and is therefore endowed with a greater translational relevance [12, 13]. This study reports that lymphodepleted D2.*mdx* mice treated with anti-FAP CAR-T cells show an infiltration of these cells in targeted organs associated with the elimination of pathological fibroblasts, a reduced expression of FAP and fibrotic markers and an improved cardiac contractility compared to untreated mice.

## Material and methods

### Mice

All procedures were performed in accordance with National and European legislations, under the license APAFIS #34549 (French Ministry of National Education, Higher Education and Research). DBA/2J (n = 4) and D2.*mdx* (DBA/2J background) (n = 97) male mice were obtained from Charles Rivers and The Jackson Laboratory, respectively. Animals were housed in the Sorbonne University animal facility under specific pathogen-free conditions, with free access to food and water, and maintained on a 12-h light/12-h dark cycle. Each cage was equipped with environmental enrichment items, including wooden chew blocks, shelters, and nesting substrates. In this study, the experimental unit is the individual animal, as *n* corresponds to the number of animals used. Our work has been reported in line with the ARRIVE guidelines 2.0.

### CAR-T cell generation

Primary murine cells were collected from spleens and lymph nodes of syngeneic D2.*mdx* mice (n = 20) following euthanasia by cervical dislocation. T cells were isolated by a negative immuno-magnetic separation (CD11b, CD11c, CD19, CD45R, CD49b, CD105, Anti-MHC-class II, and Ter-119) using the mouse Pan T Cell Isolation Kit II (Miltenyi Biotec). T cells were cultured in T cell media composed of RPMI medium 1640-GlutaMAX supplemented with 10% heat-inactivated fetal bovine serum, 50 U/mL penicillin, 50 mg/mL streptomycin, 1 mM sodium pyruvate, 10 mM HEPES and 50 mM 2-mercaptoethanol, and maintained at 37 °C and 5% CO_2_. T cells were activated in anti-CD3-coated 24-well plates, at 2 × 10^6^ cells/well in T cell media with anti-CD28 antibody and mouse IL-12. After overnight incubation, cells were expanded with 100 U/mL human IL-2 for 48 h. GFP anti-FAP CAR and GFP constructs were used for GFP anti-FAP CAR-T cell and GFP-T cell transduction, respectively. A retroviral bicistronic vector encoding a CAR composed of the anti-murine FAP (containing the scFv fragment from the specific mouse FAP antibody [clone 73.3] coupled to the human CD3ζ and CD28 cytoplasmic domains) and a reporter gene for GFP was used. Infective particles were collected from the supernatants of 293 T HEK cells transiently transfected with the retroviral vector and helper plasmids using jetPRIME® (Polyplus). T cells were then transduced by two rounds of spin-infection performed at 24 and 48 h after T cell activation using retroviral particles supplemented with polybrene. Before each injection, the transduction efficiency was assessed by flow cytometry via GFP expression and recognition of the recombinant murine FAP protein.

### In vitro functionality assays

Effectors and target cells were mixed at 1:1 to 5:1 (effector: target) ratios in 96-well plates. After 16–18 h, the expression of activation markers (PD-1, CD69) and the release of granzyme b were determined by flow cytometry. Interferon (IFN)-γ expression was assessed by ELISA (Bio-Techne).

### Lymphodepletion and CAR-T cell injection

To induce transient lymphodepletion, a single dose of cyclophosphamide (3 mg) was administered intraperitoneally to 6-month-old male D2.*mdx* mice (body weight: 25.8 ± 2.1 g). Blood samples were collected from the submandibular vein at various time points, and WBC were counted. Four days following cyclophosphamide treatment, mice received BSA (n = 8) or 10^7^ positively transduced cells (either anti-FAP CAR-T cells, n = 9 or GFP-T cells, n = 8) via the tail vein in an injected volume of 150µL. Group allocation (BSA, GFP-T cells, anti-FAP CAR-T cells) was performed on the day of injection. Treatments were prepared and coded by an independent individual, ensuring that the operators performing the injections were blinded to group identity.

### Flow cytometry and antibodies

For CAR-T cells characterization and killing assays, cells were stained with the following antibodies: anti-CD3e (clone_145-2C11; BioLegend); anti-CD8a (clone_53-6.7; BioLegend); anti-His-Tag (Cell Signaling Technology); anti-PD-1 (clone_29F.1A12; BioLegend), anti-CD69 (clone_H1.2F3; BioLegend) and anti–granzyme B (clone_QA18A28; BioLegend). For ex vivo analysis, blood was collected by the submandibular vein, and red blood cells were removed using red blood cell lysis buffer (eBioscience). Blood cell suspensions were Fc-blocked using anti-CD16/32 (clone_93; BioLegend) and stained with anti-CD3e (clone_145-2C11; BioLegend). Data were collected using a CytoFLEX LX (Beckman Coulter) and analyzed with FlowJo v10.6.2 (BD Biosciences).

### Functional cardiac evaluations

Measurements of left ventricular dimensions and volumes were performed by transthoracic echocardiography using the VEVO 3100 Imaging system (FUJIFILM VisualSonics) with an ultrasound probe MX550D from a 25 to 55 MHz frequency range. For these assessments, mice were anesthetized under isoflurane (induction with 2% and maintenance with 0.5%). All echocardiographic measurements were performed in a random order (based on the position of the cages on the rack) to avoid introducing bias into the assessments. All measurements were performed on digital loops in triplicate and used M-mode corrected cube formula. Using Vevo LAB software, data were analyzed and reviewed by a senior echocardiographist (A.H.) blindly to the treatment group. After the second echocardiography, all animals were euthanized using deep inhalation anesthesia with 5% isoflurane. The heart, which had to remain intact, *i.e.*, still beating at the time of harvesting, was collected for histological, molecular and transcriptomic analyses.

### Real-time quantitative PCR (polymerase chain reaction)

Total RNA was extracted using Trizol (Thermo Fisher Scientific) following the manufacturer’s instructions. cDNA was then synthesized from 1 μg of total RNA using the RevertAid First Strand cDNA Synthesis kit with random hexamers, according to the manufacturer’s instructions (Thermo Fisher Scientific). Gene expression was quantified by quantitative real-time PCR using the Light Cycler® 480 system (Roche Diagnostics). To prevent amplification of genomic DNA, primers were designed, when possible, to span exon-exon junctions. Two reference genes, *Hmbs* (Hydroxymethylbilane synthase) and *Gapdh* (Glyceraldehyde-3-phosphate dehydrogenase), were used for data normalization. Data were collected and analyzed on the LightCycler® 480 software (Roche). All sequences of primers used are presented in a Supplemental Table S1.

### Multiplex assay

Sera were collected from mice after centrifugation of blood sampling and stored at − 80 °C until analysis. Multiplex assay was performed using a customed 11-Plex ProcartaPlex Panel (ThermoFisher) (GM-CSF, IFN gamma, IL-1 beta, IL-2, IL-6, IL-10, IL-12p70, IL-17A (CTLA-8), IP-10 (CXCL10), MIP-1 alpha (CCL3), TNF alpha) following manufacturer’s instructions with a BioPlex 200 system (Bio-Rad).

### Histology and immunohistochemistry

Hearts were fixed by formaldehyde after dissection and incubated in sucrose gradient from 10 to 30% for 24 h. They were then frozen in liquid nitrogen-cooled isopentane. Transverse serial Sections (7 μm) of hearts were obtained using a cryostat. For GFP and PDGFRα expression, frozen sections were blocked with 5% donkey serum, then incubated overnight with primary antibodies against GFP (Invitrogen) and PDGFRα (R&D system). After washes in PBS, sections were incubated with secondary antibodies. The nuclei were stained with DAPI for 5 min. Images were captured using a Leica SP8 confocal microscope (Leica Microsystems). Quantifications and morphometric analyses were performed using the software ImageJ and blinded to treatment groups.

### Single-cell RNA-seq sample and library preparation

ScRNAseq was performed on hearts isolated from WT (DBA/2J mice, n = 2) and dystrophic D2.*mdx* mice treated either with GFP-transduced T lymphocytes (n = 5) or with GFP anti-FAP CAR-T cells (n = 5). Circulating leukocytes were labelled via intravenous injection of 2 µg anti-CD45.2 (clone_104, BioLegend) 5 min before euthanasia. Hearts were collected and digested at 37 °C with collagenase I 450U/ml, collagenase XI 125U/ml, hyaluronidase 60U/ml (all from Sigma-Aldrich) and DNAse I 60U/ml (Roche), filtered, resuspended with erythrocyte lysis buffer (Miltenyi Biotec), and viable cells were enriched using dead cell removal beads (Miltenyi Biotec). Cells were labelled with anti-MEFSK4 (Miltenyi Biotec), anti-CD45.2 (BioLegend), and each sample labelled with a TotalSeq-B Hashtag antibody (BioLegend): DBA/2J mice (n = 2): hashtag 11, 12; D2.*mdx* mice treated with GFP-transduced T lymphocytes (n = 5): hashtag 1, 4, 5, 7, 10; D2.*mdx* mice treated with anti-FAP CAR-T cells (n = 5): hashtag 2, 3, 6, 8, 9. Cells were labelled with Calcein Violet-AM (BioLegend) and Fixable Viability Dye eFluor™ 780 (ThermoFisher). Calcein( +)ViabilityDye(neg)CD45.2-PE(neg) cells were sorted using a FACS Aria II with a 100 µm nozzle and resuspended in PBS with 0.2% ultrapure BSA (ThermoFisher). ScRNA-seq libraries were generated using Chromium GEM-X Single Cell 3’ Reagent Kits v4. 35,000 cells (88.5% viability) were loaded. Libraries were prepared according to the manufacturer’s instructions and sequenced on a Novaseq (Illumina).

### Single-cell RNA-seq data analysis

Sequencing data were demultiplexed and mapped with Cell Ranger 8.0.0 (10 × Genomics) using Mouse mm10 (Ensembl 98) for the alignment. The feature–barcode matrix was analysed using Seurat v5.1.0 [14]. The HTODemux function was used to identify sample of origin, single cells and multiplets. Cells with more than 10% mitochondrial transcripts were excluded. Clustering was initially performed using the FindNeighbors and FindClusters functions with 30 PCs and a resolution of 0.8. Two clusters of dead cells (high mitochondrial transcript content) and low-quality cells (very low UMI counts) were excluded, and clustering performed again with 30 PCs and a resolution parameter of 0.4. Cellular lineages were identified based on the expression of canonical marker transcripts (*e.g. Dcn*, *Col1a1*: fibroblasts; *Myh11*: vascular smooth muscle cells; *Ptprc*, *Cd3d*: immune and T cells; *Cdh5*: endothelial cells). Enriched transcripts in fibroblast clusters were determined using the FindAllMarkers function. Fibroblast cluster proportions were determined using the dittoFreqPlot function within the dittoSeq package (https://github.com/dtm2451/dittoSeq). Gene Ontology enrichment analysis was performed using the clusterProfiler [15] v4.12.6 R package. Pseudo-bulk count matrices were generated for the cluster “Fibroblast 3” by summing gene counts from all the cells annotated as “Fibroblast 3” in each sample, and analyzed using DESeq2 version 1.44.0, [16] with an adjusted *p* value cutoff of 0.05.

### Quantification and statistical analysis

All statistical tests were performed using Prism v.9.2.0 (GraphPad). Data are expressed as means ± SEM or box and whisker plots highlighting minimum, maximum and median values. Unpaired t-test, Mann–Whitney, Kruskal–Wallis, one-way ANOVA, and two-way ANOVA tests were used as indicated in individual figure legends, using post hoc Tukey’s and Dunn’s tests for multiple comparison correction. All statistical tests were two-tailed with a significance level of 0.05. ns, not significant; *p < 0.05, **p < 0.01, ***p < 0.001 and ****p < 0.0001. The required sample size for animal studies was calculated based on a two-sided test with a significance level (α) of 0.05 and a statistical power (1 − β) of 0.80, assuming a standard deviation (σ) of 10.

## Results

### Anti-FAP CAR-T cells are activated when co-cultured with FAP + target cells

T cells were isolated from spleens and lymph nodes of D2.*mdx* mice by a negative immuno-magnetic selection (see Methods) and activated using CD3/CD28 antibodies and Interleukin (IL)-2 for 2 days. In parallel, a retroviral bicistronic vector encoding an anti-murine FAP (scFv fragment from the mouse FAP antibody coupled to the human CD3ζ/CD28 cytoplasmic domains) and a reporter gene for Green Fluorescent Protein (GFP) was constructed. Isolated T cells were then retrovirally transduced to express either GFP alone (GFP-T cells) or both GFP and the CAR receptor (anti-FAP CAR-T cells). Prior to in vivo studies, we first assessed the rate of lymphocyte transduction by flow cytometry measuring GFP and CAR expression. Subsequently, the cytotoxicity of transduced cells against FAP + target cells was evaluated by flow cytometry and ELISA (Figure S1). The transduction rates were 85.3% ± 8.6% GFP and 0.3% ± 0.3% CAR expression for the GFP-T cells (n = 6) and 79.9% ± 8.8% GFP and 80.3% ± 7.1% CAR (n = 6) for the anti-FAP CAR-T cells. Anti-FAP CAR-T cells or untransduced T cells (effector cells) were then co-cultured with FAP + or FAP- 293 T HEK cells (target cells). Following 16 h of exposure to FAP + target cells, anti-FAP CAR-T cells significantly overexpressed CD69 and PD-1, two classic activation-dependent T cell indicators, [17] by a factor of 3.75 ± 1.25 (n = 4) and of 1.39 ± 0.13 (n = 3), respectively, compared with effector cells alone, without target cells. They also released the cytotoxic factor granzyme b (1.68 ± 0.13-fold, n = 3), which is implied in apoptosis induction, [18] and almost 1000-fold greater amounts of interferon γ (*Ifn-γ)*, as measured by ELISA, compared to effector cells alone, without target cells (Figure S1). The combination of these two metrics (transduction efficiency and cytokine secretion) meets the regulatory requirements for CAR-T cell analytical testing (https://www.fda.gov/vaccines-blood-biologics/guidance-compliance-regulatory-information-biologics). Of note, the genetically-induced overexpression of FAP in the target 293 T HEK cells, required for assessing the functional activation of the anti-FAP CAR-T cells, does not deter from the relevance of their therapeutic use in the clinics for mitigating fibrosis given the increased natural expression of FAP in our murine disease model and the transcriptomics reported in DMD patients (GSE38417 dataset).

### Anti-FAP CAR-T cells detection in vivo is associated with FAP downregulation in targeted organs

**Fig. 1:**
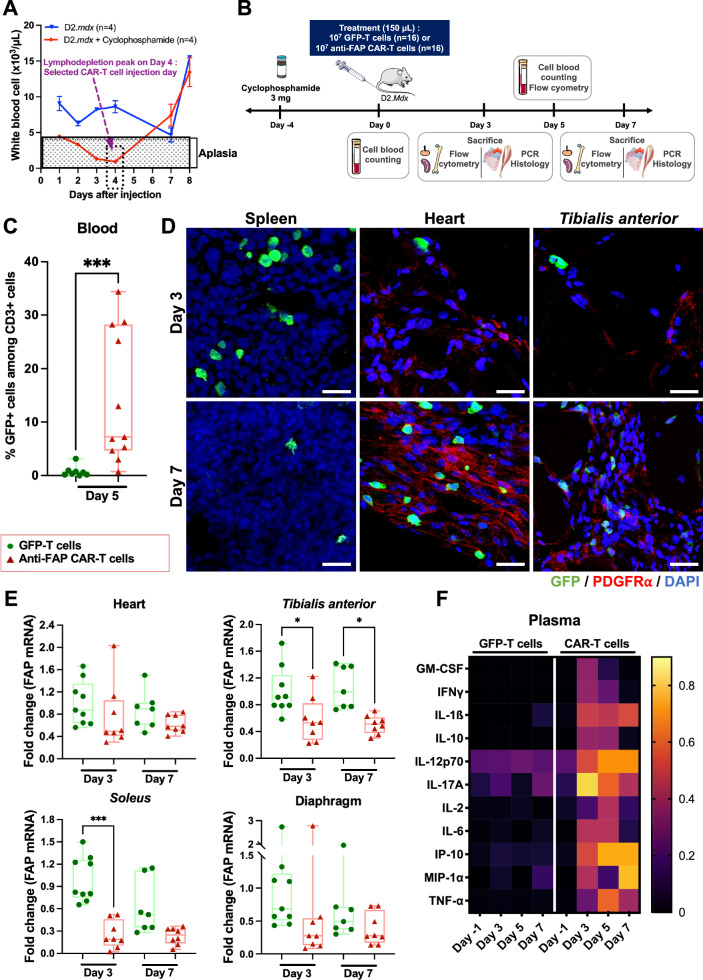
Anti-FAP CAR-T cells following a cyclophosphamide-based lymphodepletion are found in fibrotic areas and induce FAP downregulation in the heart. **A** Follow-up of white blood cells (WBC) count by regular blood sampling via the submandibular vein in D2*.mdx* mice following cyclophosphamide treatment. **B** Experimental set-up of pharmacokinetics experiments, including GFP or Anti-FAP CAR-T cells-treated D2.*mdx* mice. For each condition, mice were sacrificed at days 3 and 7. Figure was created with BioRender.com. **C** At day 5 post treatment, proportion of GFP + cells detection among CD3 + cells by flow cytometry. Box and whisker plots highlight minimum, maximum and median values. ***p < 0.001 (Mann–Whitney test). **D** Immunofluorescence of GFP + cells (green) and a marker of fibrosis (PDGFRα, red) in spleen, heart and *tibialis anterior* sections in the treated condition at days 3 & 7 post-treatment. (Scale bars = 25 µm). **E** Fold change of murine FAP mRNA measured by PCR at days 3 and 7 post injection in heart, *tibialis anterior*, *soleus* and diaphragm. Fold change was calculated relative to the condition of control cells (GFP-T cells) at day 3. Box and whisker plots highlight minimum, maximum and median values. (Kruskal–Wallis test; post-hoc multiple comparisons, Dunn’s test). **F** Heatmap representing fold change of various cytokines measured in plasma by Luminex at different timepoints: day -1 (baseline, one day prior to treatment) or days 3, 5, 7 post-treatment. Fold change was calculated relative to maximum values. Absolute cytokine concentrations are presented in Table S3

To comply with clinical practice, a prior lymphodepletion was implemented to prevent anti-CAR immune responses and facilitate in vivo expansion and activity of CAR-T cells [19]. After conducting several optimization experiments, a single intraperitoneal injection of cyclophosphamide (3 mg) was found to be safe and effective in temporarily reducing the white blood cell (WBC) count, which reached its nadir 4 days after injection (Fig. 1A). Mice receiving cyclophosphamide fully recovered in one week. Based on these results, we determined the optimal timing of CAR-T cell treatment was 4 days post-lymphodepletion. We then conducted pharmacokinetic experiments, repeated twice independently, which entailed a single intravenous injection of either 10^7^ anti-FAP CAR-T cells or GFP-T cells (Fig. 1B). Anti-FAP CAR-T cells were detected in blood by flow cytometry 5 days post-injection. The proportion of GFP + cells among CD3 + cells was 14.30% ± 3.72% in the anti-FAP CAR-T cells group, compared to 0.76% ± 0.36% in the GFP-T controls (Fig. 1C). Anti-FAP CAR-T cells were also present in lymphoid organs, including spleen, lymph nodes and bone marrow (Figure S2). Confocal microscopy analysis of GFP expression confirmed these findings, showing that anti-FAP CAR-T were robustly detected in the spleen compared to GFP-T cell treatment at day 3, with a decrease by day 7 (Fig. 1D).

We then extended our findings to the targeted organs, the heart and *tibialis anterior* (Fig. 1D). Anti-FAP CAR-T cells co-localized with fibrotic regions identified by Platelet-Derived Growth Factor Receptor Alpha (PDGFRα) staining. Two-photon microscopy with second-harmonic generation (SHG) to visualize collagen confirmed the vicinity of CAR-T cells and PDGFRα staining (Figure S3). The density of anti-FAP CAR-T cells increased between days 3 and 7 in both the heart and *tibialis anterior* (Fig. 1D, Table S2). Compared to GFP-T cells, anti-FAP CAR-T treatment significantly increased GFP DNA detection in the heart by 19-fold at day 3 and 53-fold at day 7. In the *tibialis anterior*, the increase was even more pronounced, with 58-fold at day 3 and 358-fold at day 7 (Figure S4). This increase in anti-FAP CAR-T cell presence was paralleled by a decrease in endogenous FAP expression in the heart, *tibialis anterior* as well as in the diaphragm and *soleus*, as assessed by PCR at days 3 and 7 (Fig. 1E).

### Anti-FAP CAR-T cells activation is associated with a downregulation of fibrotic markers

To document treatment activation in organs of interest, we measured several cytokines, including GM-CSF, IFNγ, IL-1β, IL-10, IL-12p70, IL-17a, IL-2, IL-6, IP-10, MIP-1α and TNFα at baseline (one day prior to treatment) and on days 3, 5 and 7 post-treatment in plasma using Luminex. Compared to mice injected with GFP-T control cells, those treated with anti-FAP CAR-T cells showed elevated plasma levels of several cytokines (Fig. 1F and Table S3). Whether this observation is suggestive of a murine equivalent of the Cytokine Release Syndrome (CRS) [20] remains speculative; whatsoever, the downregulation of the majority of these markers by day 7, coupled with the lack of overt clinical signs of toxicity, and particularly the absence of body weight loss (Supplemental Table S4), in CAR-T cell-treated mice was reassuring with regard to safety. These results were confirmed by PCR in targeted organs (Figure S5). The cardiac expression of *Acta2* and *Itgbl1* was also down-regulated after anti-FAP CAR-T cell therapy, compared with controls, with about a 55% decrease at day 3 (0.14 ± 0.06 *vs* 0.06 ± 0.02 for *Itgbl1*; 5.37 ± 0.59 *vs* 2.31 ± 0.45 for *Acta2*), followed by about a 20% decrease at day 7 (0.06 ± 0.01 *vs* 0.04 ± 0.01 for *Itgbl1*; 5.85 ± 0.41 *vs* 4.71 ± 0.52 for *Acta2*), as measured by PCR.

### Anti*-*FAP CAR-T cells therapy improves cardiac function

**Fig. 2:**
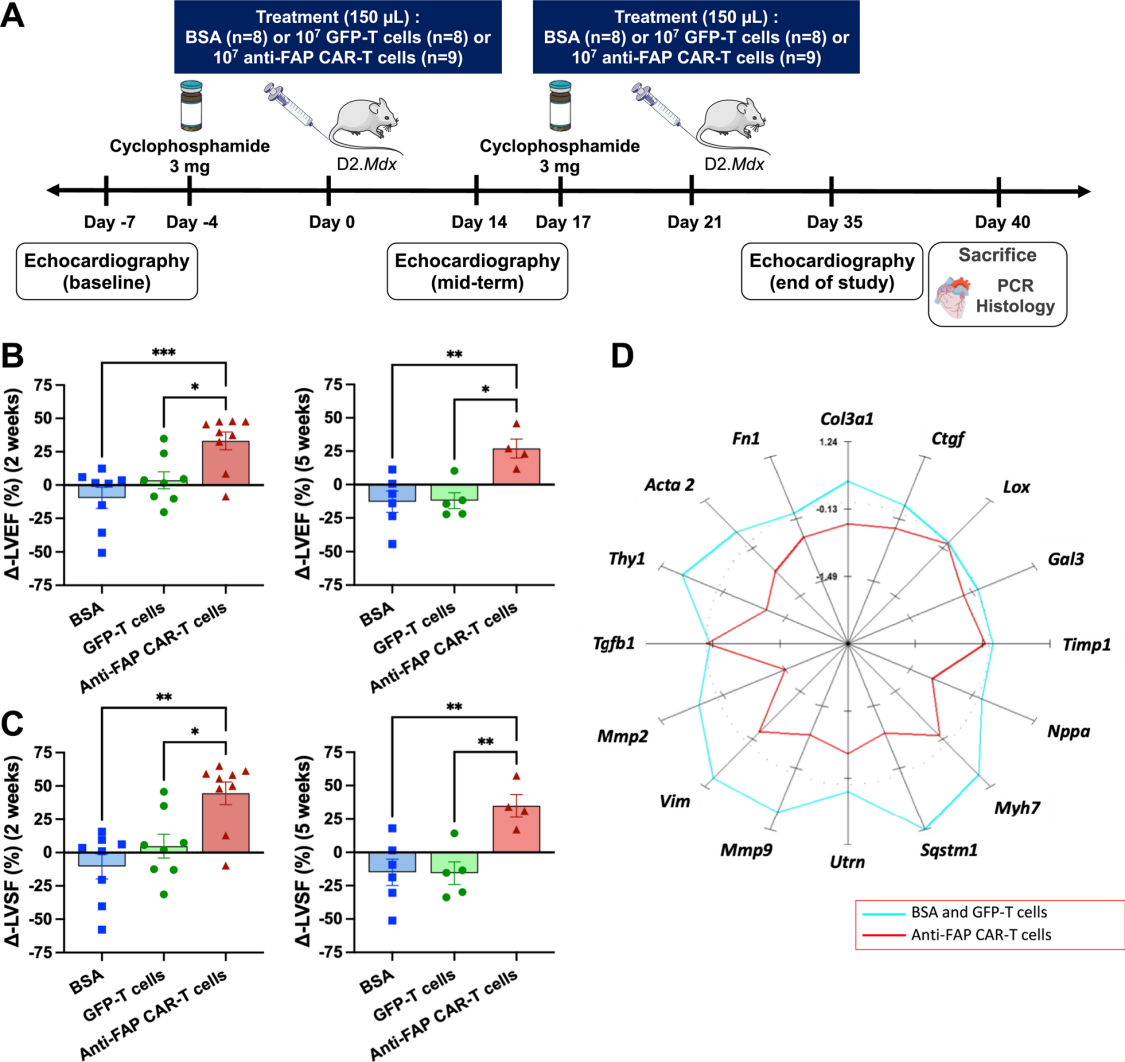
Anti-FAP CAR-T cells treatment improves cardiac function. **A** Experimental set-up for functional assessment experiments in D2.*mdx* mice. Cyclophosphamide was intraperitoneally injected twice and followed by 2 intravenous injections of the different treatments, 2 weeks apart. Echocardiography was performed 1 week before treatment (baseline) and 2 and 5 weeks thereafter. **B, C** Changes in left ventricular ejection fraction (LVEF) **(B)** and changes in left ventricular shortening fraction (LVSF) **(C)** relative to baseline measured by echocardiography with M-mode corrected cube formula at 2- and 5-weeks post treatment. Data are presented as means ± SEM. *p < 0.05, **p < 0.01, ***p < 0.001 (Ordinary one-way ANOVA; post-hoc multiple comparisons, Tukey’s test). **D** Radar representation of standardized values of fibrotic and heart failure gene expression of CAR-T cells and control conditions (data from GFP-T cells and BSA groups were pooled together). Full names of all the studied genes are found in Table S2. The figure includes material from SMART Servier medical Art (https://smart.servier.com/) under a Creative Commons Attribution 4.0 license (CC BY 4.0)

These pharmacokinetic data prompted us to undertake another set of experiments focused on the cardiac functional outcome of anti-FAP CAR-T cells, using bovine serum albumin (BSA) and genetically modified T cells, here by the GFP reporter gene, as negative controls. Four days after lymphodepletion, 6-month D2.*mdx* mice were intravenously injected with either a single dose or two doses (3 weeks apart) of anti-FAP CAR-T cells, GFP-T cells and BSA and were followed-up by echocardiography (Fig. 2A). This age group was selected because it corresponds to a greater degree of cardiac fibrosis and dysfunction compared with younger mice [21]. At 2 weeks, anti-FAP CAR-T cell significantly improved left ventricular ejection fraction (LVEF), with a change from baseline of 33.14% ± 6.66% (n = 9) compared to 3.66% ± 6.41% (n = 8) and -9.59% ± 7.95% (n = 8) in the GFP-T cells and BSA groups, respectively. The benefit was maintained at 5 weeks post-treatment with an LVEF change from baseline in anti-FAP CAR-T-injected mice of 26.98% ± 7.10% (n = 4) *vs.* -12.03% ± 5.93% (n = 5) and -12.84% ± 7.98% (n = 6) in the GFP-T cells and BSA groups, respectively (Fig. 2B). The changes in LV fractional shortening (LVFS) featured similar patterns (Fig. 2C). Individual trajectories of LVEF and LVSF over time for each animal are presented in Figures S6–7. Following sacrifice, we measured several fibrotic and heart failure markers in these same hearts by PCR. As shown in Fig. 2D, gene expression revealed distinct profiles of these markers between the anti-FAP CAR-T cells group and controls (BSA and GFP-T cells combined), suggesting their global downregulation in the treated group. The observation of an improved cardiac function along with the above-mentioned downregulation of endogenous FAP levels evidenced by PCR tends to suggest that even relatively low levels of FAP expression were efficiently hit by the anti-FAP CAR-T cells treatment.

### Anti-FAP CAR-T cells prevent expansion of pathogenic fibroblasts in D2.*mdx* hearts

**Fig. 3:**
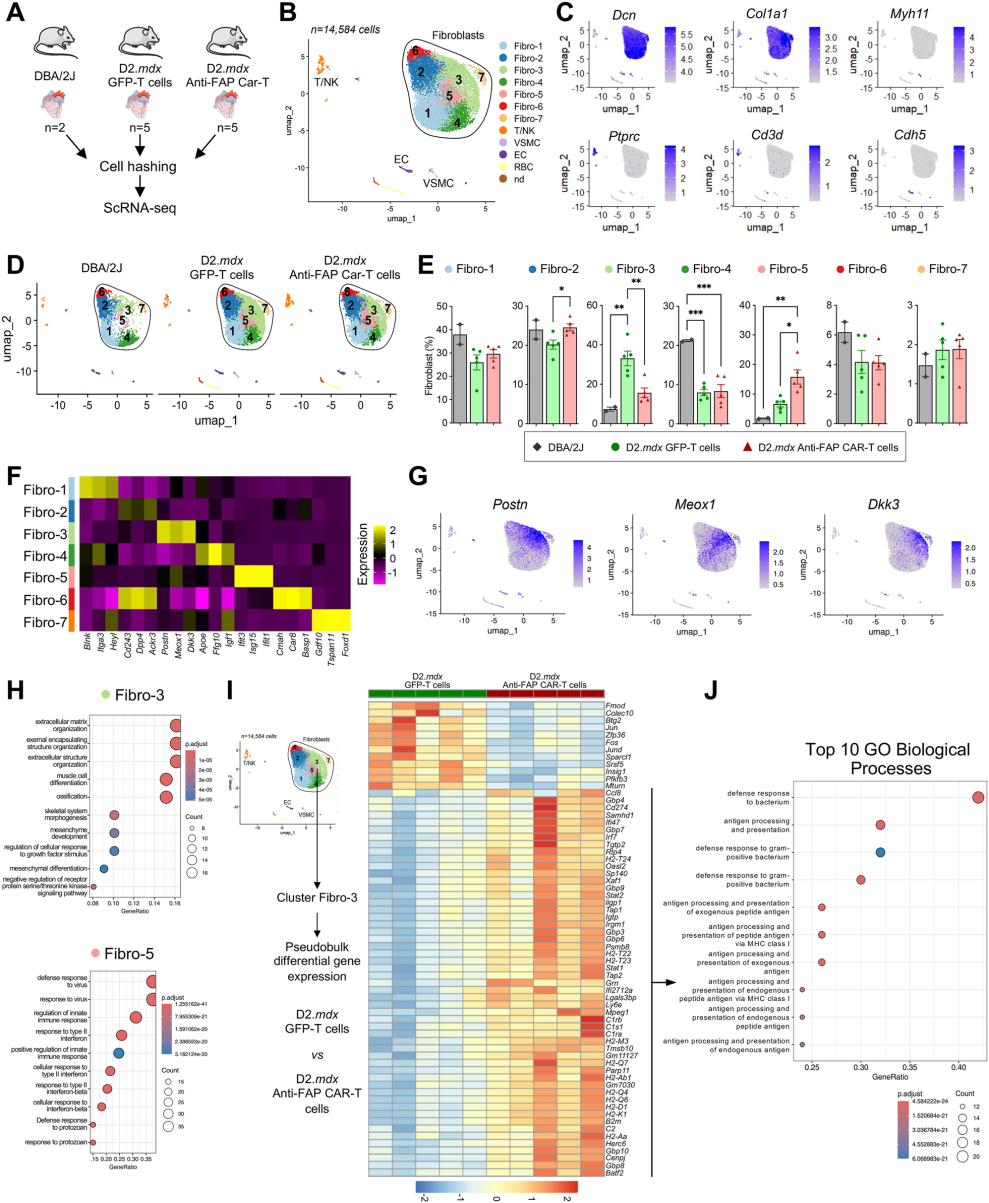
Anti-FAP CAR-T cells alter fibroblast subsets in D2.*mdx* mouse hearts. **A** Experimental setup; **B** UMAP plot of recovered cells with clusters annotated and color coded; **C** Expression of lineage markers (*Dcn*, *Col1a1*: fibroblasts; *Myh11*: vascular smooth muscle cells; *Ptprc*, *Cd3d*: immune and T cells; *Cdh5*: endothelial cells) used to identify cell types projected on the UMAP plot; **D** UMAP plot split according to experimental condition; **E** Proportion of fibroblast clusters within total fibroblasts; **F** Heatmap of average expression of selected marker genes in fibroblast clusters; **G** Expression of pro-fibrotic fibroblast markers *Postn*, *Meox1* and *Dkk3* projected on the UMAP plot; **H** Gene ontology "biological process" analysis in the fibroblast clusters 3 and 5 (top 10 enriched terms); **I** Pseudobulk differential gene expression analysis in cells of the Fibroblast cluster 3 in GFP-T cells *vs* anti-FAP CAR-T cells treated D2.*mdx* mice; **J** Gene ontology "biological process" analysis performed on upregulated genes in fibroblast cluster 3 in anti-FAP CAR-T cells treated D2.*mdx* mice as shown in (I) Fibro: fibroblast; T/NK: T cells/Natural Killer cells; VSMC: vascular smooth muscle cells; EC: endothelial cells; RBC: red blood cells; nd: not determined; GO: gene ontology. The figure includes material from SMART Servier medical Art (https://smart.servier.com/) under a Creative Commons Attribution 4.0 license (CC BY 4.0)

In a mechanistic perspective, we performed single-cell RNA-sequencing of non-cardiomyocyte viable cardiac cells in healthy DBA/2J (n = 2), GFP-T cells (n = 5) and anti-FAP CAR-T cells (n = 5)-treated D2.*mdx* mice. Individual samples were labelled with hashtag antibodies [22] and pooled in one scRNA-seq library (Fig. 3A). 14,584 cells were used for analysis after quality control filtering (Fig. 3B), and we identified major cellular lineages based on the expression of characteristic marker transcripts including endothelial cells (*Cdh5*), vascular smooth muscle cells (*Myh11*), immune cells (*Ptprc*, comprising *Cd3d*^+^ T cells), and fibroblasts (*Col1a1*, *Dcn*) (Fig. 3C). Fibroblasts comprised 7 clusters (Fibro-1 to Fibro-7) with clear distribution shifts between healthy DBA/2J and diseased D2.*mdx* hearts (Fig. 3D, E), and with specific gene expression profiles (Fig. 3F–H). In particular, the cluster Fibro-3 had a gene expression profile characteristic of disease-associated fibroblasts in cardiac fibrosis contexts, with enrichment in markers such as *Postn* (encoding periostin) [23] and *Meox1* [24] (Fig. 3F–G), and enriched biological processes such as “extracellular matrix organization” (Fig. 3H). Proportions of the Fibro-3 population within the total fibroblast pool were significantly increased in GFP-T cells-treated D2.*mdx* mice compared to healthy DBA/2J controls, an effect that was abrogated by anti-FAP CAR-T cell treatment, indicating a preferential ablation of this subset endowed with a pathogenic, pro-fibrotic signature (Fig. 3D, E). Differential gene expression analysis further showed that anti-FAP CAR-T cell treatment induced a pro-inflammatory profile within the Fibro-3 cluster (Fig. 3I, J). The Fibro-5 cluster displayed a pro-inflammatory interferon response profile with characteristic marker transcripts (*Isg15*, *Ifit1*, *Ifit3*) and enriched biological processes (*e.g.* “response to interferon beta”) and was significantly expanded in anti-FAP CAR-T cell-treated D2.*mdx* mice (Fig. 3D, G). Altogether, these results indicate that in D2.*mdx* hearts, anti-FAP CAR-T cells induce specific shifts in cardiac fibroblast phenotypes, particularly characterized by the dampening of the activated pro-fibrotic Fibro-3 cluster.

## Discussion

The main findings of this study are that anti-FAP CAR-T cells, injected after an effective lymphodepletion, (i) expand in blood, home to lymphoid organs and traffic to the heart, diaphragm and skeletal muscles, (ii) ablate FAP + pathogenic fibroblasts in these tissues, and (iii) improve cardiac function for up to 5 weeks after treatment.

While correcting dystrophin gene mutations remains the cornerstone of DMD treatment, alleviating the associated fibrosis, which occurs early in the disease course, is also clinically relevant for both mitigating the negative effects of fibrosis on cardiac function and overcoming the fibrosis-associated barrier that may impede delivery of gene products [25]. This is well illustrated by the observation that the anti-FAP CAR-induced mitigation of skeletal muscle fibrosis is associated with an increase in adeno-associated virus (AAV) microdystrophin gene transfer [26]. Overall, the extent and location of fibrosis are reported to be robust predictors of adverse cardiac events [27]. 

In our in vivo experiments, prior lymphodepletion allowed anti-FAP CAR-T cells to expand [28] and to be unequivocally identified in the targeted organs, while their presence in lymphoid organs was only transient, supporting their preferential homing towards FAP-expressing sites. In preliminary experiments in which lymphodepletion was omitted (data not shown), CAR-T cells still improved function but to a lesser degree than following cyclophosphamide conditioning, likely reflecting their smaller number and/or shorter persistence in the circulation [29]. It is however unlikely that the use of cyclophosphamide, which can promote the development of cardiac fibrosis, [30] may have confounded the results since all mice, both the controls and the CAR-T treated ones, received the same lymphodepletion drug regimen. Likewise, the activity of the anti-FAP CAR-T cells was apparently not impeded by the potential cyclophosphamide-induced IL-7 and IL-15-mediated proliferation of residual lymphocytes [31].

So far, the upregulation of FAP in cardiac tissue has been described in patients with hypertrophied or dilated cardiomyopathies and in mice subjected to repeated Angiotensin II/Phenylephrine infusions [3]. The present study extends the relevance of FAP overexpression to the completely different setting of DMD, as shown by PCR data. The primary mechanism whereby CAR-T cells improved cardiac function, whose deterioration is a major driver of mortality in DMD patients, [8] is likely the ablation of the pathogenic fibroblasts. This assumption is primarily based on single-cell sequencing which demonstrated the significant decrease of a pathogenic fibrogenic fibroblast cluster after anti-FAP CAR-T cell treatment, compared with untreated controls. Interestingly, a similar cluster of pathogenic fibroblasts has been identified in the hearts of patients with advanced heart failure, [32] thereby supporting the potential benefit of its therapeutic targeting. This assumption is consistent with the observation that knock-out of periostin which is expressed in this fibrogenic cluster has been shown to improve skeletal muscle structure and function in a mouse model of muscular dystrophy [33]. In line with the observation that the expression of FAP is associated with that of other fibrosis-related genes [34], the myocardial tissue levels of several matrix fibrogenesis markers (*Mmp2*, *Mmp9*, *Vim*, *Thy1*, and *Acta2*) were reduced and this mitigation of the fibrotic burden may have contributed to improve cardiac function by decreasing myocardial stiffness [35]. Of note, in a mouse model of bleomycin-induced pulmonary fibrosis, the ability of anti-FAP CAR-T cells (generated in vivo by CD5-targeted lipid nanoparticles) to reverse pulmonary fibrosis has been found associated with a similar decrease in the pro-fibrotic fibroblast clusters [36] Additionally, cardiac fibroblasts can affect cardiomyocyte function through paracrine signaling. The knock-down of Integrin beta-like 1 (*Itgbl1*), a functional mediator involved in the fibroblast-cardiomyocyte crosstalk, has been shown to blunt fibrosis and improve cardiac function [37]. In our study, we observed a trend towards decreased expression of *Itgbl1* in heart. Of note, the relationship between cardiomyocyte number and heart function may not be strictly linear as the loss of an even small fraction of cardiomyocytes can profoundly decrease heart function [38]. Assuming the reverse may be true, even a modest reduction in the fibrotic burden positively affecting cardiomyocyte integrity could contribute to improve inotropism if cardiomyocytes are not only viewed as contractile elements but also as sources of paracrine factors helping to preserve myocardial tissue at the whole organ level [38]. These data may thus explain why the removal of FAP-expressing activated fibroblasts, even if partial, resulted in better preservation of LVEF and LVFS with a parallel decrease in heart failure biomarkers (*i.e. Nppa, Myh7*). The link between the ablation of FAP + cells and the improvement in cardiac function is further supported by the above-mentioned clinical study suggesting that FAP-targeted imaging may stand as a predictive marker of cardiac dysfunction [4]. Put together, our data support the occurrence of cell-to-cell and cell-to-extracellular matrix communication networks [11] which might amplify the signals initially conveyed by anti-FAP CAR-T cells and broaden their spectrum to the mitigation of the whole fibrotic process and the attendant preservation of cardiac function.

This study has obviously several limitations. First, even though the cardiac functional benefits coupled with the lack of clinical signs of on-target off-tissue toxicity despite the transient release of inflammatory markers, point towards a favourable efficacy/safety ratio, additional risk analysis studies are required to more comprehensively characterize the therapeutic index of anti-FAP CAR-T cells in this specific DMD indication. This concern stems from the previous observations of lethal bone toxicity and cachexia in tumour-bearing mice after adoptive immunotherapy targeting FAP [39]. However, these complications were likely facilitated by a more aggressive protocol entailing a broader irradiation-induced lymphodepletion (with slower recovery kinetics), a two-fold higher dose of CAR-T cells and the stimulation of their persistence by subsequent repeated injections of Il-2. While such a harsh protocol makes sense in the context of cancer where complete eradication of the tumoral burden is mandatory, it can likely be less aggressive in the DMD context where an only partial abrogation of fibrosis can yet have cardio-protective effects, thereby allowing to favourably uncouple the mechanisms underlying efficacy from those involved in toxicity. An additional reassuring observation related to safety comes from a pre-printed publication reporting, in a metabolic dysfunction-associated steatohepatitis model, that an anti-FAP CAR (delivered by lipid nanoparticles) significantly reduced liver fibrosis without signs of hepatotoxicity or significant changes in body weight [40]. Second, studies should also focus on longer term experiments to assess whether the benefits of anti-FAP CAR-T cells are sustained over time and, if not, whether it is appropriate to consider repeat dosing in light of some specific safety issues like an immune response against the CAR (except if another construct is used) or the risks of a new lymphodepletion (Guidance Document from the FDA, FDA-2021-D-0404). However, given that fibrosis is the end-result of a self-perpetuating genetically-induced myofiber injury, inflammation and dysregulated repair mechanisms, [41] one can speculate that if a gene therapy product could successfully correct the mutation while part of the already present fibrosis is eradicated by CAR-T cells, the pathological process could then be stopped. Third, while the current study focused on the effects of anti-FAP CAR -T cells on cardiac function, it is now equally important to extend the investigation to skeletal muscle function in DMD. Finally, we acknowledge that the ex vivo production of CAR-T cells, with the associated requirement for a lymphodepletion, is hardly conceivable in these young DMD patients, for both ethical and safety reasons. However, our study still carries a translational potential in that the reported proof-of-concept data pave the way to an in vivo programming of lymphocytes by a FAP-encoding messenger RNA encapsulated in lipid nanoparticles, which has already been shown effective in an hypertensive cardiomyopathy model [42] and would likely address most of the bottlenecks still associated with ex vivo CAR-T cell manufacturing.

Despite these limitations, if one puts together the in vitro evidence for lymphocyte activation, the downregulation of FAP mRNA in the target organs and the more sensitive single-cell sequencing-based finding of a decrease in the fibrosis-associated FAP-expressing cluster of pathogenic fibroblasts, the data point to the ability of anti-FAP CAR-T cells to have efficiently hit their target and provide some mechanistic grounds to the improvement in cardiac function. Thus, this first proof-of-principle study introduces anti-FAP CAR-T adoptive therapy as a potential new player in the arsenal of anti-fibrotic strategies complementing DMD gene therapy and suggests that it could efficiently contribute to mitigate cardiac dysfunction, which remains the leading cause of death in DMD patients [5, 8].

## Supplementary Information

Supplementary Material 1.

## Data Availability

All data reported in this paper will be shared by the lead contact upon request. Single-cell RNA-sequencing raw (fastq files) and processed (10 × Genomics Cell Ranger matrices) data have been deposited in BioStudies: accession number S-BSST1818 ( https:/doi.org/10.6019/S-BSST1818). The code used for single-cell RNA-sequencing analysis is provided as supplementary files (R notebook html files). Any additional information required to reanalyze the data reported in this paper is available from the lead contact upon request.
