# Supplementary material for: CAR-T cells targeting fibroblast activation protein eliminate pathological fibroblasts and preserve cardiac function in a Duchenne Muscular Dystrophy murine model
Source: Stem Cell Res Ther. 2026 Apr 30;17:222. doi: 10.1186/s13287-026-05025-1 (PMC13277170; doi:10.1186/s13287-026-05025-1)
Supplement: Supplementary file 1 — Supplementary Material 1. [file 13287_2026_5025_MOESM1_ESM.pdf]

Supplementary Information

Supplemental Figures

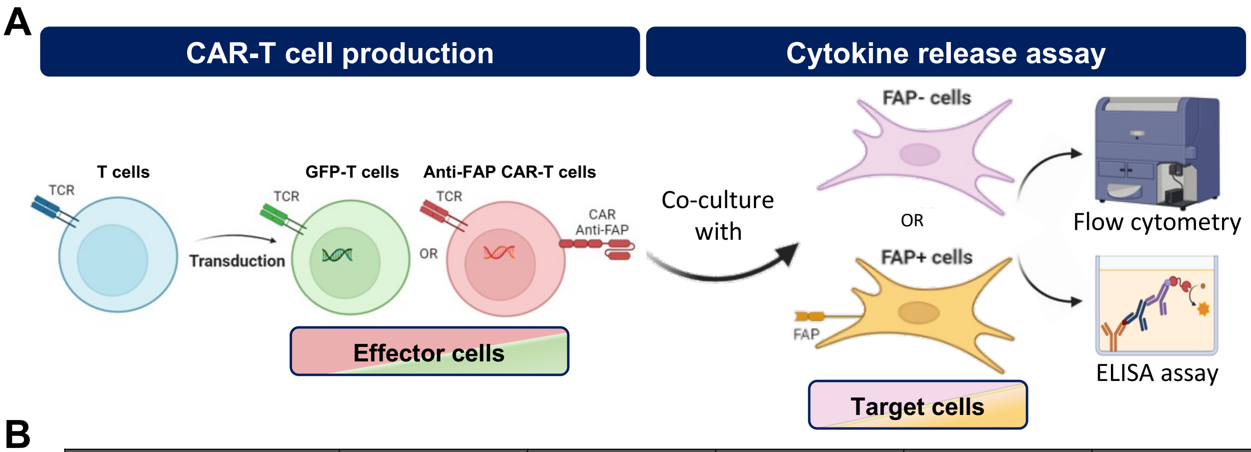

B

Sample Name	CD3+ T cells	CD4+ T cells	CD8+ T cells	GFP+ cells	CAR+ cells
T cells	99.2% ± 0.7%	61.6% ± 9.2%	23.7% ± 5.6%	0.6% ± 0.8%	0.4% ± 0.4%
GFP-T cells	99.1% ± 0.6%	64.6% ± 4.9%	22.0% ± 4.2%	85.3% ± 8.6%	0.3% ± 0.3%
Anti-FAP CAR-T cells	99.0% ± 0.7%	65.5% ± 4.6%	23.4% ± 3.8%	79.9% ± 8.8%	80.3% ± 7.1%

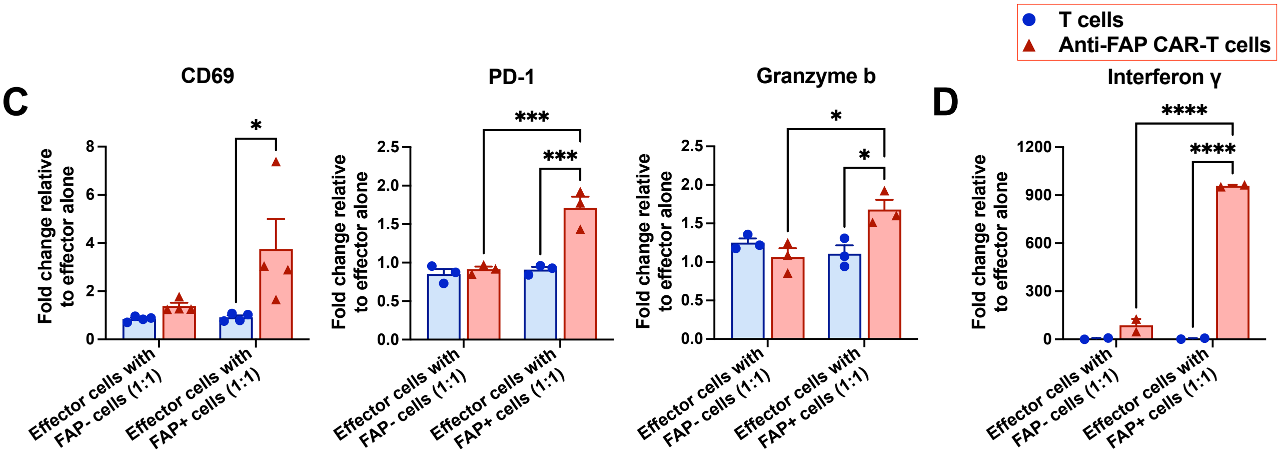

Figure S1. (Related to Figure 1) Anti-FAP CAR-T cells transduction enables a specific activation when co-cultured with FAP+ cells. **(A)** Schematic representation of the three cell types and *in vitro* experimental set-up. Figure was created with BioRender.com. **(B)** Characterization of isolated and transduced T cells (GFP-T cells and anti-FAP CAR-T cells) by CD3, CD4, CD8, GFP and CAR expression by flow cytometry (n=6). Results are presented as means ± SEM. **(C)** Fold change of CD69 (n=4), PD-1 (n=3) and granzyme b (n=3) measured by flow cytometry after co-culture of effectors (either Anti-FAP CAR-T cells or T cells) on target cells (either FAP+ or FAP- cells) for 16 hours. Fold change was calculated relative to the condition of effector cells alone without target cells. Data are presented as means ± SEM. *p<0.05, **p<0.01, ****p<0.0001 (Two-way ANOVA; post-hoc multiple comparisons, Tukey's test). **(D)** Fold change of interferon γ concentration (n=2) measured by ELISA after co-culture of effectors (either CAR-T cells or T cells) on target cells (either FAP+ or FAP- cells) for 16 hours. Fold change was calculated relative to the condition of effector cells alone, without target cells. Data are presented as means ± SEM. *p<0.05, **p<0.01, ****p<0.0001 (Two-way ANOVA; post-hoc multiple comparisons, Tukey's test).

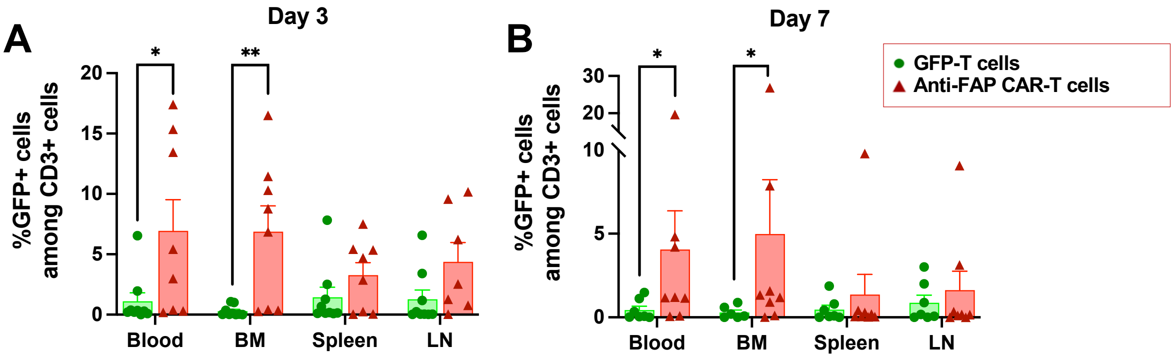

Figure S2. (Related to Figure 1) Anti-FAP CAR-T cells are present in all the lymphoid organs studied, but transiently. At days 3 and 7 post treatment, proportion of GFP+ cells detection among CD3+ cells by flow cytometry. Data are presented as means ± SEM. **p*<0.05, ***p*<0.01 (Mann-Whitney test).

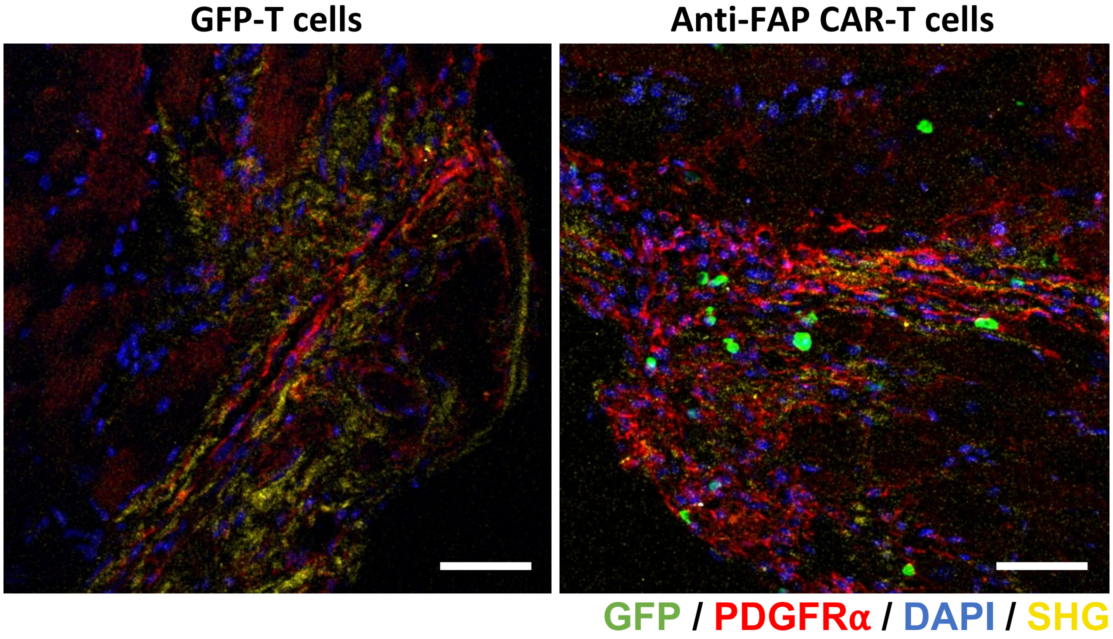

Figure S3. (Related to Figure 1) Anti-FAP CAR-T cells are co-localized in fibrotic areas detected by Second Harmonic Generation (SHG). SHG as a marker of collagen (yellow) coupled with immunofluorescence co-staining GFP + cells (green) and a marker of fibrosis (PDGFRα, red) in heart. (Scale bars:25 µm).

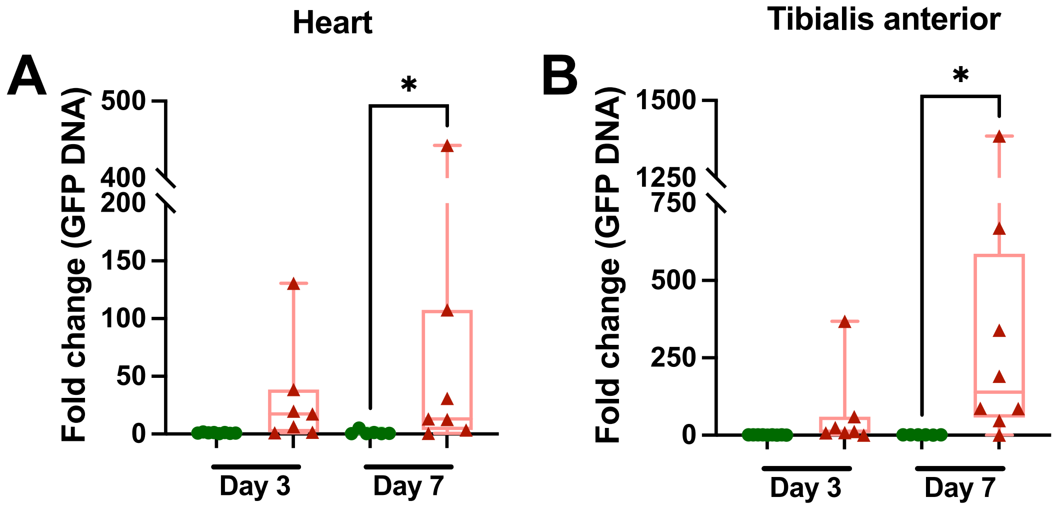

Figure S4. (Related to Figure 1) Anti-FAP CAR-T cells are detected in targeted organs by PCR. Fold change of GFP DNA detection measured by PCR at days 3 and 7 post injection in heart and TA. Fold change was calculated relative to the condition of control cells (GFP-T cells) at day 3. Box and whisker plots highlight minimum, maximum and median values. ***p*<0.01 (Kruskal-Wallis test; post-hoc multiple comparisons).

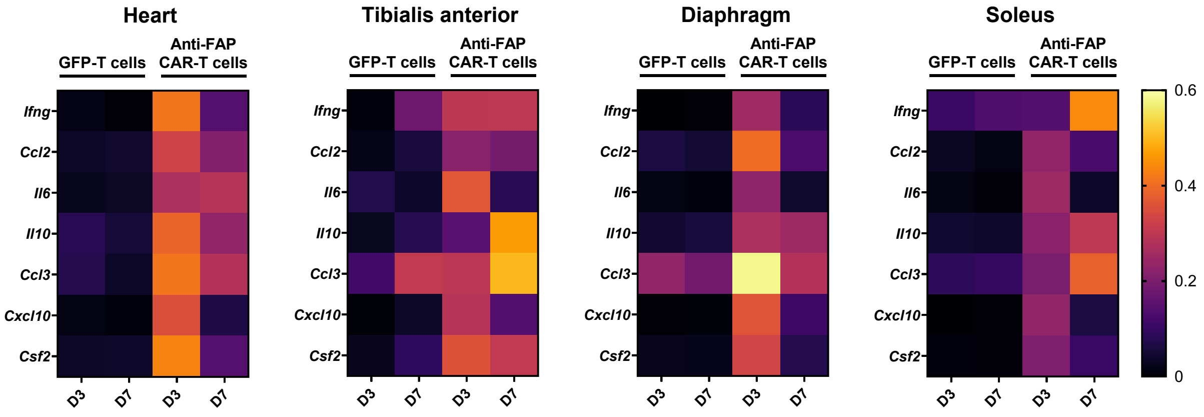

Figure S5. (Related to Figure 1) Anti-FAP CAR-T cells induced local cytokines upregulation reflecting their activation in the targeted organs. Heatmap representing fold change of various cytokines measured in heart by PCR at days 3 and 7 post injection. Fold change was calculated relative to the condition of control cells (GFP-T cells) at day 3. Full names of all the studied genes are found in Table S1.

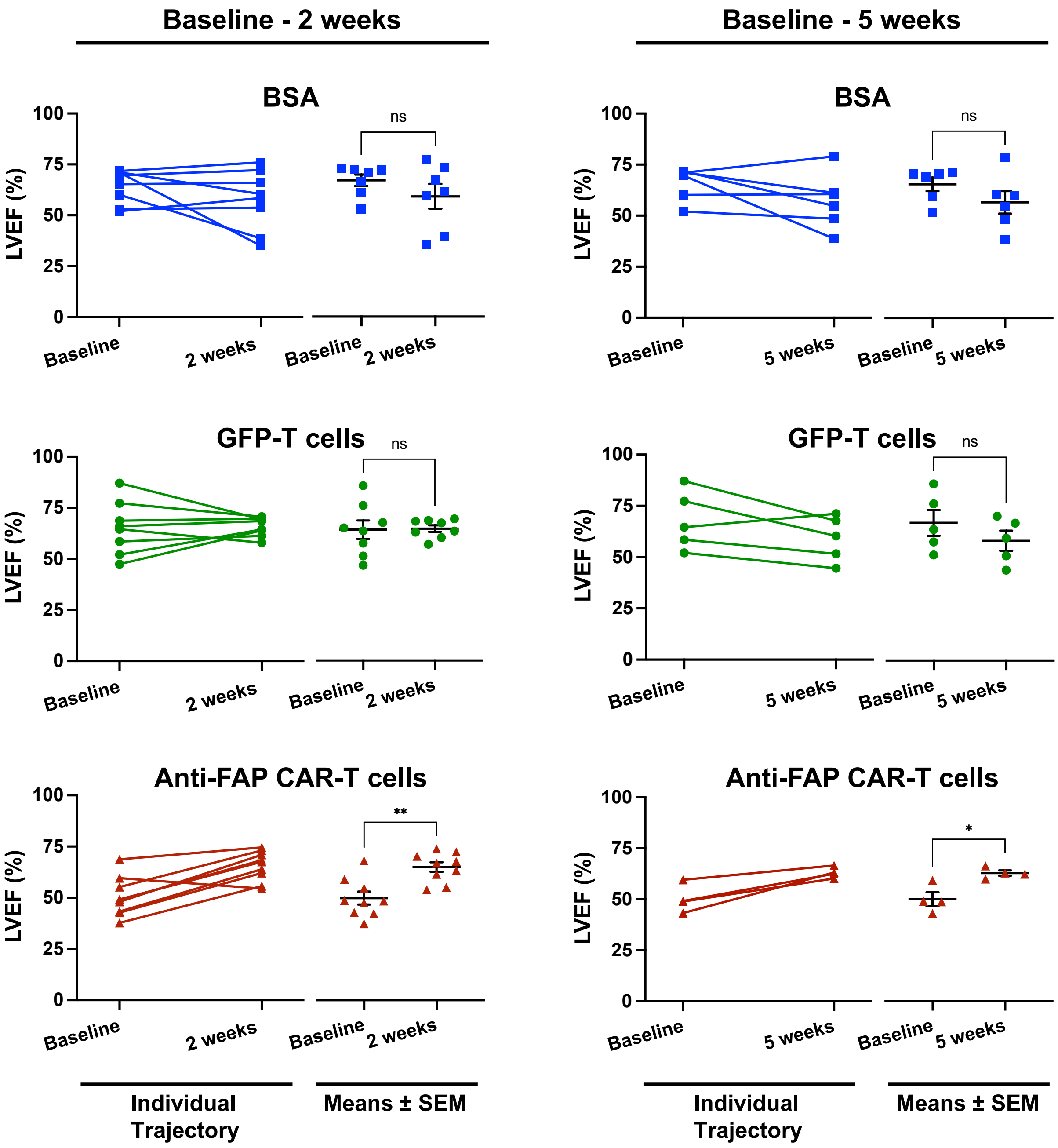

Figure S6. (Related to Figure 2) Functional assessment (LVEF) of anti-FAP CAR-T cells treatment. Echocardiography was performed 1 week before treatment (baseline) and 2 and 5 weeks thereafter. The figure represents individual trajectories of LVEF from baseline to the final study point for each animal. Note the significant improvement from baseline to 2- and 5 weeks post-treatment in anti-FAP CAR-T-treated animals. Values are presented as percentages. *p<0.05, **p<0.01 (Paired t-test or Wilcoxon matched-pairs signed rank test).

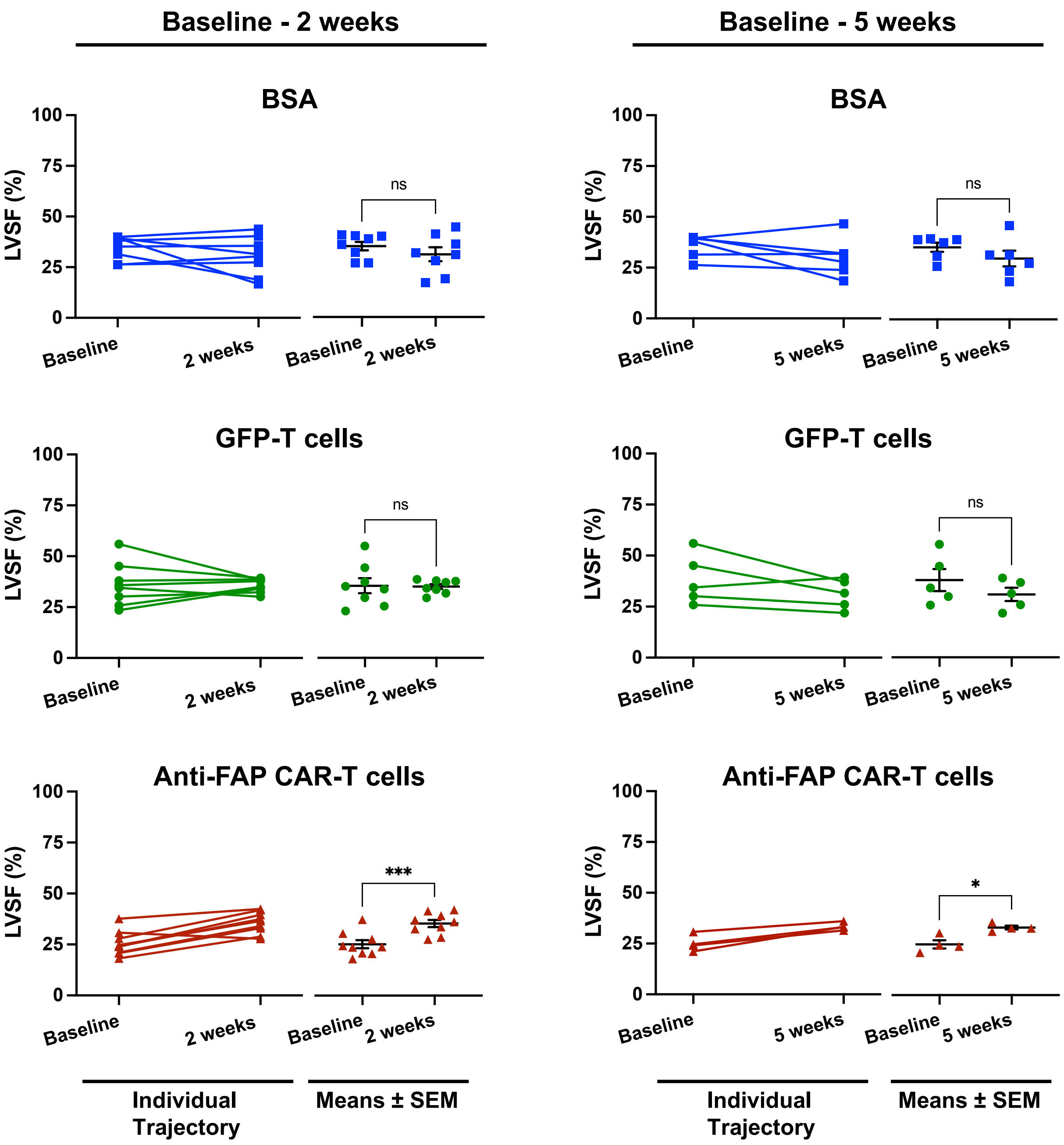

Figure S7. (Related to Figure 2) Functional assessment (LVSF) of anti-FAP CAR-T cells treatment. Echocardiography was performed 1 week before treatment (baseline) and 2 and 5 weeks thereafter. The figure represents individual trajectories of LVSF from baseline to the final study point for each animal. Note the significant improvement from baseline to 2- and 5 weeks post-treatment in anti-FAP CAR-T-treated animals. Values are presented as percentages. *p<0.05, ***p<0.001 (Paired t-test or Wilcoxon matched-pairs signed rank test).

Supplemental Tables

Table S1. (Related to Figure 1&2) List of all the studied genes and sequences of associated primers.

Genes	Full name gene	Forward (5' – 3')	Reverse (5' – 3')
**Treatment**			
Gfp	Green Fluorescent Protein	GACGTAAACGGCCACAAGTT	GAACTTCAGGGTCAGCTTGC
**Fibrosis**			
Fap	Fibroblast Activation Protein	CACCTGATCGGCAATTTGTG	CCCATTCTGAAGGTCGTAGATGT
Col1a1	Collagen Type I Alpha 1 Chain	ATTCCCGTTCGAGTACGGAA	CTCGATCTCGTTGGATCCCT
Col3a1	Collagen Type III Alpha 1 Chain	AAAGAGGATCTGAGGGCTCG	TCACCTCCAACTCCAGCAAT
Ctgf	Connective Tissue Growth Factor	ACTCTGCCAGTGGAGTTCAA	TTACGCCATGTCTCCGTACA
Lox	Lysyl Oxidase	TGAACAAATAGCGGAGGGGC	GAAAGCGCACAGAGTCTGGA
Gal3	Galectin 3	CCCTTTGAGAGTGGCAAACC	TGATCCCCAGTTGGCTGATT
Sqstm1	Sequestosome 1	AGCTGCCCTATACCCACATC	CAGCCCCGATGTCGTAATTC
Nppa	Natriuretic Peptide A	ATATTGGAGCAAATCCTGTG	GTCTAGCAGGTTCTTGAAAT
Myh7	Myosin Heavy Chain 7	AGGTGTGCTCTCCAGAATGG	CAGCGGCTTGATCTTGAAGT
Timp1	TIMP Metallopeptidase Inhibitor 1	ATATCCGGTACGCCTACACC	AAAGCTCTTTGCTGAGCAGG
Mmp2	Matrix Metallopeptidase 2	CAGGGCACCTCCTACAACAG	CAGTGGACATAGCGGTCTCG
Mmp9	Matrix Metallopeptidase 9	GTCTTTGAGTCCGGCAGACA	CCAGTACCAACCGTCCTTGA
Vim	Vimentin	TGCCTCTGCCAACCTTTTCT	GGTGTCAACCAGAGGAAGTGACT
Utrn	Utrophin	CACTATGACCCCTCCCAGTC	CGCTTCCTGTTGTAGAGCTG
Thy1	Thy-1 Cell Surface Antigen	TGCAGGTTCAGTCAGGGAAT	CCTGCACGGACTTAGATCCT
Acta2	Actin Alpha 2	AGATCAAGATCATTGCCCCTCC	TTGTGTGCTAGAGGCAGAGC
Fn1	Fibronectin 1	CGAAGAGCCCTTACAGTTCCA	ATCTGTAGGCTGGTTCAGGC
Tgfb1	Transforming Growth Factor, Beta 1	GTCACTGGAGTTGTACGGCA	GGGCTGATCCCGTTGATTTC
Itgbl1	Integrin Subunit Beta Like 1	CACGATGGTCTCATTTGCAC	CATTTCCATTCCATCCATCC
**On-target cytokines**			
Ifn-γ	Interferon-γ	GTTTGAGGTCAACAACCCACA	ACTCCTTTTCCGCTTCCTGA
Ccl2	Chemokine Ligand 2	CAGGTGTCCCAAAGAAGCTG	ACCTTAGGGCAGATGCAGTT
Il-6	Interleukin 6	TCTCTGCAAGAGACTTCCATCC	AAGTCTCCTCTCCGGACTTG
Il-10	Interleukin 10	GGCGCTGTCATCGATTTCTC	GCCTTGTAGACACCTTGGTC
Ccl3	Chemokine Ligand 3	CCCTCTGTCACCTGCTCAAC	GGGGTGTCAGCTCCATATGG
Cxcl10	CXC motif Chemokine Ligand 10	TTTCTGCCTCATCCTGCTGG	CATTCTCACTGGCCCGTCAT
Cfs2	Colony Stimulating Factor 2	AAGAAGCCCTGAACCTCCTG	AAATTGCCCCGTAGACCCTG

Table S2. (Related to Figure 1) Relative quantification of GFP+ cells from different sections of hearts and tibialis anterior at different timepoints.

Conditions	Mouse Indentification Code	Organs	GFP+ cells number	Heart area (pixels²)	Ratio
**Day 3**					
GFP-T cells	1088	Heart	3.50	107.10	0.03
		Tibialis	n.d.	40.38	-
	1079	Heart	n.d.	98.20	-
		Tibialis	0.13	38.40	0.00
Anti-FAP CAR-T cells	1090	Heart	198.00	101.90	1.94
		Tibialis	2.25	50.61	0.04
	1072	Heart	176.50	102.80	1.72
		Tibialis	0.50	38.32	0.01
	1063	Heart	63.80	106.80	0.60
		Tibialis	n.d.	45.50	-
**Day 7**					
GFP-T cells	1107	Heart	0.50	108.20	0.00
		Tibialis	n.d.	38.48	-
	1080	Heart	1.80	86.60	0.02
		Tibialis	0.83	34.26	0.02
Anti-FAP CAR-T cells	1091	Heart	1616.50	94.60	17.08
		Tibialis	501.13	43.57	11.50
	1101	Heart	132.70	80.20	1.65
		Tibialis	82.63	49.65	1.66

Table S3. (Related to Figure 1) Absolute cytokine concentration measured in plasma by Luminex at different timepoints: day -1 (baseline, one day prior to treatment), days 3, 5 and 7 post-treatment.

Cytokines	Parameters	Baseline (Day -1)		Day 3		Day 5		Day 7	
		GFP-T cells	Anti-FAP CAR-T cells	GFP-T cells	Anti-FAP CAR-T cells	GFP-T cells	Anti-FAP CAR-T cells	GFP-T cells	Anti-FAP CAR-T cells
GM-CSF (pg/ml)	Mean	0.32	0.58	0.14	38.15	0.47	12.62	0.07	1.55
	Std. Deviation	0.27	0.52	0.19	62.93	0.00	17.31	0.00	0.38
	Std. Error of Mean	0.09	0.20	0.08	36.33	0.00	10.00	0.00	0.27
IFNgamma (pg/ml)	Mean	0.34	0.73	0.61	1970.45	2.25	1404.84	0.63	232.90
	Std. Deviation	0.30	0.82	0.58	3114.72	2.75	1062.97	0.13	202.54
	Std. Error of Mean	0.10	0.31	0.26	1798.28	1.945	613.71	0.09	143.22
IL-1 beta (pg/ml)	Mean	0.00	0.04	0.00	0.82	0.00	0.77	0.16	0.89
	Std. Deviation	0.00	0.07	0.00	0.86	0.00	0.45	0.23	0.35
	Std. Error of Mean	0.00	0.03	0.00	0.49	0.00	0.26	0.16	0.245
IL-10 (pg/ml)	Mean	0.43	0.52	1.08	60.48	0.43	57.78	0.77	7.75
	Std. Deviation	0.18	0.43	0.58	93.24	0.37	55.32	0.47	0.87
	Std. Error of Mean	0.06	0.16	0.26	53.83	0.26	31.94	0.335	0.615
Il-12p70 (pg/ml)	Mean	0.15	0.16	0.16	0.36	0.19	0.47	0.16	0.47
	Std. Deviation	0.03	0.03	0.03	0.20	0.01	0.17	0.00	0.31
	Std. Error of Mean	0.01	0.01	0.01	0.12	0.01	0.10	0.00	0.22
IL-17A (pg/ml)	Mean	0.59	0.71	1.11	4.70	0.32	3.38	1.55	2.46
	Std. Deviation	0.25	0.26	0.80	0.92	0.08	2.14	1.82	0.36
	Std. Error of Mean	0.08	0.10	0.36	0.53	0.05	1.24	1.29	0.25
IL-2 (pg/ml)	Mean	0.46	0.85	0.46	2.85	0.73	6.22	0.17	2.19
	Std. Deviation	0.46	0.61	0.28	1.06	0.91	7.38	0.23	0.03
	Std. Error of Mean	0.15	0.23	0.12	0.61	0.65	4.26	0.17	0.02
IL-6 (pg/ml)	Mean	4.89	2.41	18.39	198.67	0.99	198.26	23.14	50.85
	Std. Deviation	5.94	2.64	14.94	224.22	1.12	182.18	11.53	36.49
	Std. Error of Mean	1.98	1.00	6.68	129.46	0.80	105.18	8.16	25.81
IP-10 (pg/ml)	Mean	14.63	16.78	37.35	211.83	31.04	301.88	33.26	302.23
	Std. Deviation	10.09	10.53	22.23	181.11	14.22	124.28	15.85	33.24
	Std. Error of Mean	3.36	3.98	9.94	104.56	10.06	71.75	11.21	23.51
MIP-1alpha (pg/ml)	Mean	0.00	0.00	0.96	6.29	0.00	2.59	2.30	11.35
	Std. Deviation	0.00	0.00	1.46	7.65	0.00	2.28	0.97	0.18
	Std. Error of Mean	0.00	0.00	0.65	4.42	0.00	1.31	0.685	0.13
TNF-alpha (pg/ml)	Mean	1.38	1.59	1.93	21.22	1.41	47.52	2.27	31.34
	Std. Deviation	0.83	0.76	0.94	19.21	1.90	32.51	2.06	2.23
	Std. Error of Mean	0.28	0.29	0.42	11.09	1.345	18.77	1.46	1.58

Table S4. Body weight of the mice at baseline and 5 weeks after treatment, in the different groups.

Conditions	n	Mean	Std. Deviation	Std. Error of Mean	95% CI
**Baseline (Body Weight) (g)**					
BSA	6	26.70	1.25	0.51	[25.39 ; 28.01]
GFP-T cells	5	25.96	2.40	1.07	[22.98 ; 28.94]
Anti-FAP CAR-T cells	4	26.58	1.73	0.86	[23.83 ; 29.32]
**5 weeks post-treatment (Body Weight) (g)**					
BSA	6	25.60	2.42	0.99	[23.06 ; 28.14]
GFP-T cells	5	24.80	1.39	0.62	[23.08 ; 26.52]
Anti-FAP CAR-T cells	4	26.55	1.97	0.98	[23.42 ; 29.68]
**Changes in body weight relative to baseline (%)**					
BSA	6	-4.23	5.76	2.35	[-10.28 ; 1.81]
GFP-T cells	5	-4.05	7.04	3.15	[-12.79 ; 4.71]
Anti-FAP CAR-T cells	4	-0.08	3.78	1.89	[-6.10 ; 5.95]
